# Comparison of post-operative three-dimensional and two-dimensional evaluation of component position for total knee arthroplasty

**DOI:** 10.1186/s43019-021-00106-2

**Published:** 2021-07-13

**Authors:** Osamu Tanifuji, Tomoharu Mochizuki, Hiroshi Yamagiwa, Takashi Sato, Satoshi Watanabe, Hiroki Hijikata, Hiroyuki Kawashima

**Affiliations:** 1 Division of Orthopedic Surgery, Niigata University Graduate School of Medical and Dental Sciences, 1-757 Asahimachi-dori Chuo-ku, Niigata City, Niigata, 951-8510 Japan; 2 Department of Orthopedic Surgery, Saiseikai Niigata Hospital, Niigata, Japan; 3 Department of Orthopedic Surgery, Niigata Medical Center, Niigata, Japan

**Keywords:** Knee, Osteoarthritis, Total knee arthroplasty

## Abstract

**Purpose:**

The purpose of this study was to evaluate the post-operative three-dimensional (3D) femoral and tibial component positions in total knee arthroplasty (TKA) by the same co-ordinates’ system as for pre-operative planning and to compare it with a two-dimensional (2D) evaluation.

**Materials and methods:**

Sixty-five primary TKAs due to osteoarthritis were included. A computed tomography (CT) scan of the femur and tibia was obtained and pre-operative 3D planning was performed. Then, 3D and 2D post-operative evaluations of the component positions were performed. KneeCAS (LEXI, Inc., Tokyo, Japan), a lower-extremity alignment assessment system, was used for the 3D post-operative evaluation. Standard short-knee radiographs were used for the 2D post-operative evaluation. Differences between the pre-operative planning and post-operative coronal and sagittal alignment of components were investigated and compared with the results of the 3D and 2D evaluations.

**Results:**

According to the 3D evaluation, the difference between the pre-operative planning and actual post-operative sagittal alignment of the femoral component and the coronal and sagittal alignments of the tibial component were 2.6° ± 1.8°, 2.2° ± 1.8° and 3.2° ± 2.4°, respectively. Using the 2D evaluation, they were 1.9° ± 1.5°, 1.3° ± 1.2° and 1.8° ± 1.4°, making the difference in 3D evaluation significantly higher (*p* = 0.013, = 0.003 and < 0.001). For the sagittal alignment of the femoral component and the coronal and sagittal alignment of the tibial component, the outlier (> ± 3°) ratio for the 3D evaluation was also significantly higher than that of the 2D evaluation (*p* < 0.001, = 0.009 and < 0.001).

**Conclusions:**

The difference between the pre-operative planning and post-operative component alignment in the 3D evaluation is significantly higher than that of the 2D, even if the same cases have been evaluated. Two-dimensional evaluation may mask or underestimate the post-operative implant malposition. Three-dimensional evaluation using the same co-ordinates’ system as for pre-operative planning is necessary to accurately evaluate the post-operative component position.

## Introduction

Component position in total knee arthroplasty (TKA) is critical in determining post-operative outcomes. Generally, although it is typically evaluated through computed tomography (CT) scans or X-rays of the knee, some studies have reported issues with accuracy of using standard knee radiography to evaluate post-operative component position [1–5]. Furthermore, it is impossible to assess this using the same co-ordinates’ system as pre-operative planning using standard CT only. Therefore, it is difficult to accurately evaluate the differences between pre-operative planning and post-operative component alignment.

We performed pre-operative three-dimensional (3D) planning based on a whole-leg CT scan and evaluated the post-operative component position using the same co-ordinates’ system as pre-operative planning [6, 7]. In this way, since the same co-ordinates’ system as for pre-operative planning was used, it was possible to evaluate a strict component position error against the pre-operative planning. The purpose of this study was to evaluate the differences between two-dimensional (2D) evaluation and 3D evaluation of the post-operative component positions.

## Materials and methods

### Study subjects

**Fig. 1:**
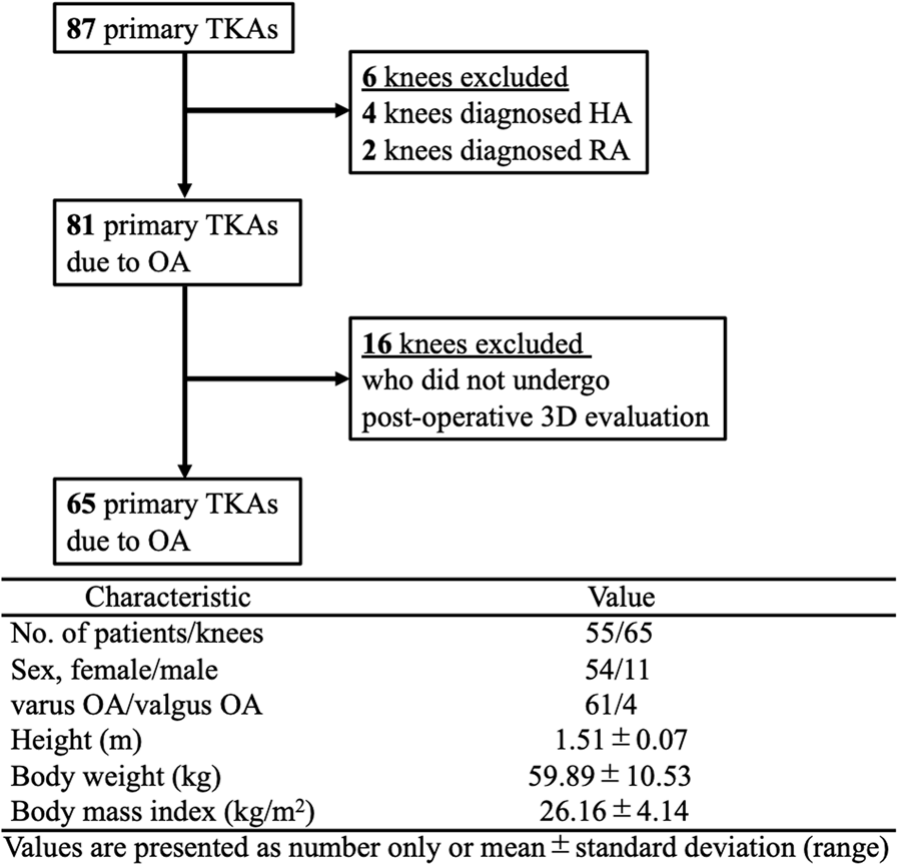
Patient demographics and flow chart of the study. *OA* osteoarthritis, *HA* haemophilic arthropathy, *RA* rheumatoid arthritis

Total knee arthroplasty (TKA) is performed for patients with tricompartmental osteoarthritis (OA) and/or severe flexion contracture that cannot be treated with unicompartmental knee arthroplasty or osteotomy, such as high tibial osteotomy, at our institution. From April 2013 to July 2019, 67 patients (87 knees) underwent primary TKA using Evolution® (MicroPort Orthopaedics; Arlington, TN, USA) at our institution. This implant was used as the primary TKA in all patients except in those with severe valgus deformities. Of those consecutive cases, we excluded patients who were pre-operatively diagnosed with rheumatoid arthritis or haemophilic arthropathy. The patients who did not undergo computerised radiography (CR) for 3D analysis after surgery were also excluded. Thus, this study assessed 65 primary TKAs due to osteoarthritis of the knee in 55 patients (female, 54 knees; male, 11 knees) with a mean age of 72.8 ± 9.5 years (range 40–84 years) (Fig. 1). This study included young patients in their 40s (three knees). Regarding these patients, all three knees had undergone cartilage-related surgery in their teens or 20s and had severe tricompartmental OA at the timing of TKA. This retrospective study followed a protocol that had been approved by the Investigational Review Boards of our institution (2015–2351). All subjects provided informed consent prior to participating in the study.

### Pre-operative planning and surgery

**Fig. 2:**
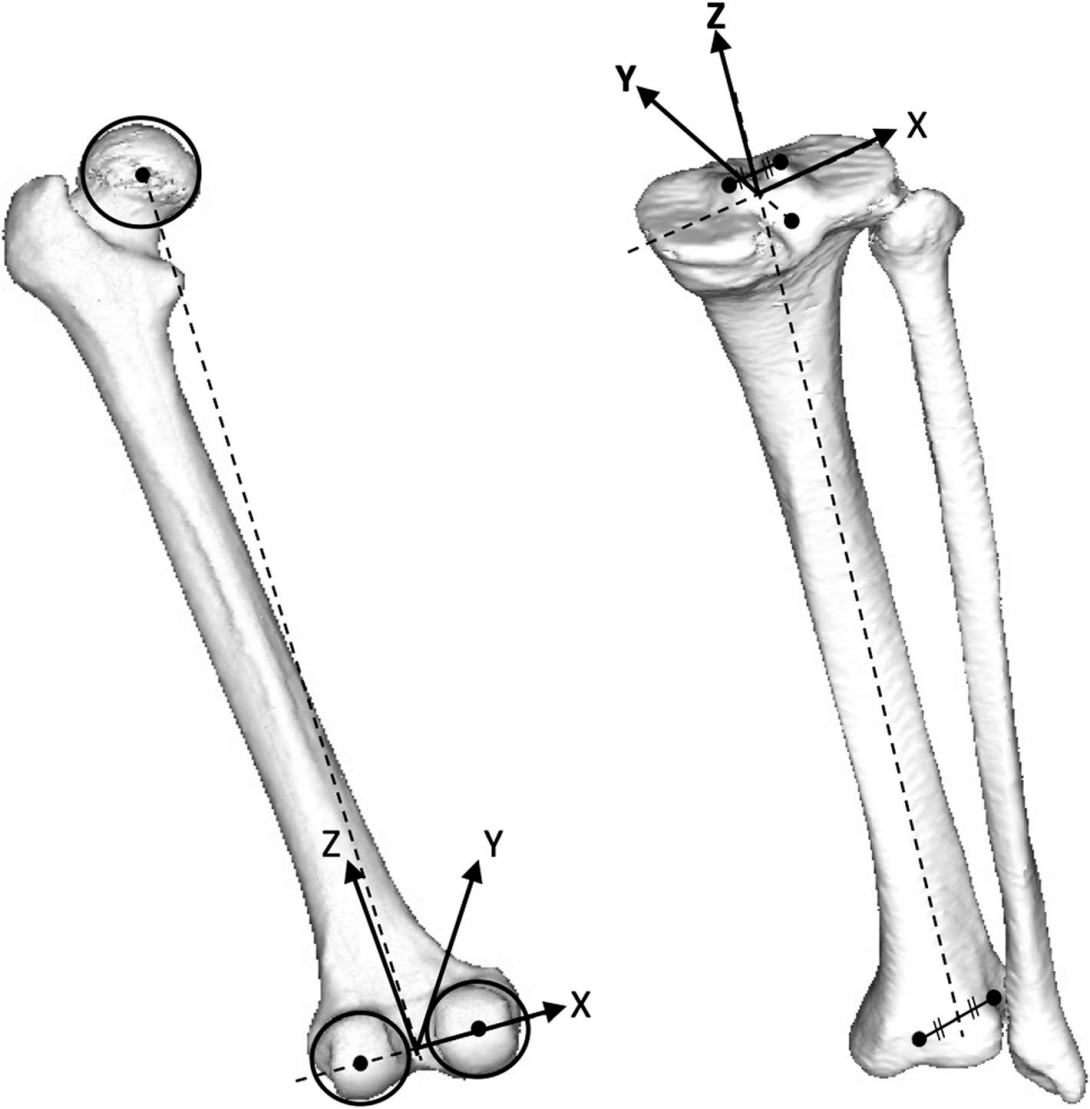
The femoral and tibial co-ordinates’ systems were constructed referencing several bony landmarks

**Fig. 3:**
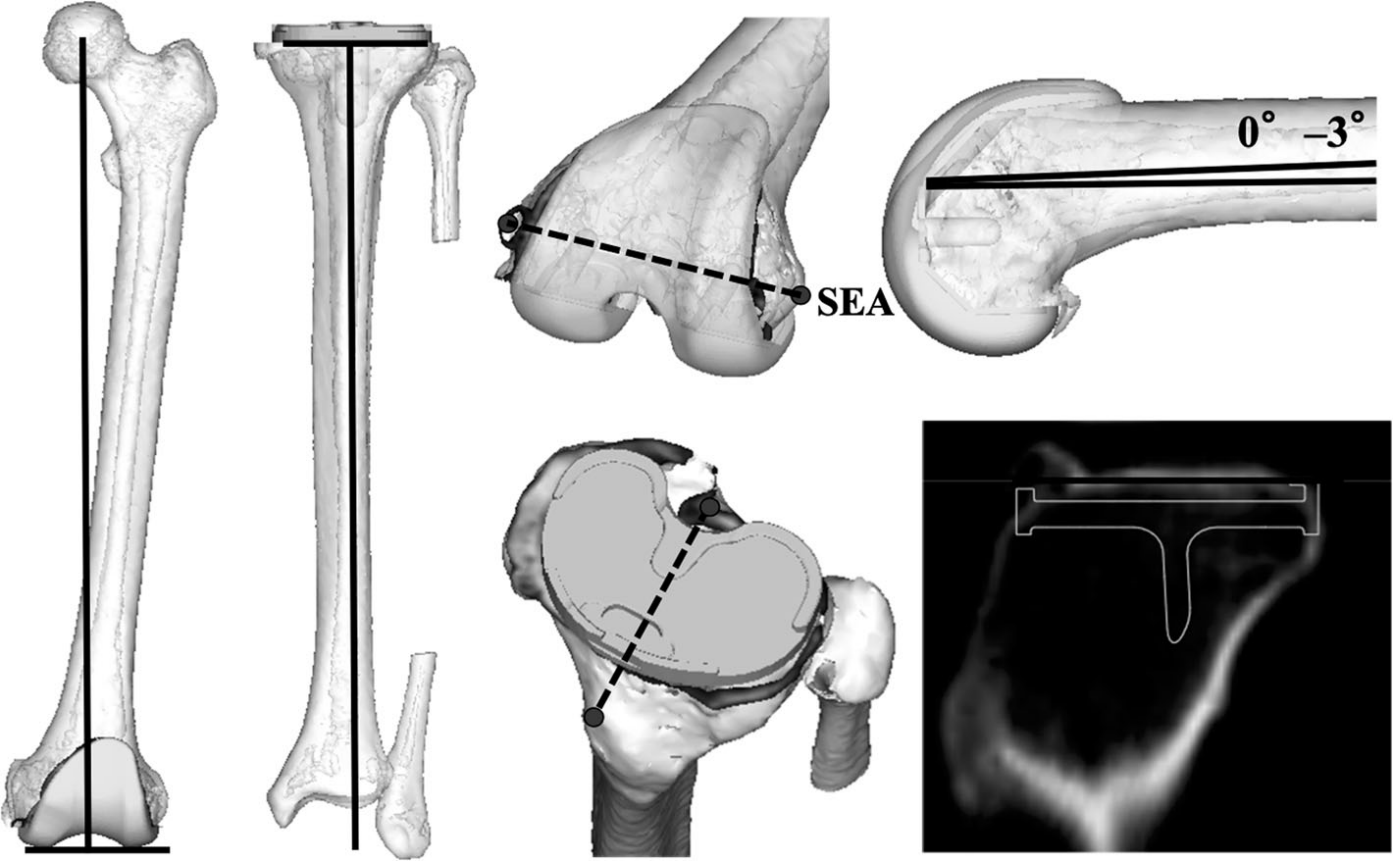
Pre-operative three-dimensional (3D) planning was performed by reading implant computer-aided design (CAD) data. *SEA* surgical epicondylar axis

A CT scan of the femur and tibia was obtained for each subject using a SOMATOM Sensation 16 (Siemens, Munich, Germany) with a 1-mm interval. Data from the CT scan were used to build a 3D digital model of the bones using ZedView (LEXI, Tokyo, Japan) visualisation and modelling software, with anatomical co-ordinates to reference several bony landmarks [7–9]. The geometric centre axis (GCA), i.e. the line connecting the centres of the spheres representing the medial and lateral posterior femoral condyles was defined as the femoral x-axis. The origin of the co-ordinates’ system was defined as the midpoint between the centres of these posterior femoral condylar spheres. The femoral z-axis was defined as being perpendicular to the x-axis and in a plane formed by the x-axis and a line connecting the femoral origin and the centre of the femoral head. The femoral y-axis was defined as the cross product of the z-axis and x-axis. The tibial z-axis was defined by a line connecting the midpoint of the tibial eminence and the midpoint of the medial and lateral superior poles of the talar dome. The tibial y-axis was defined as a perpendicular line drawn from the medio-lateral centre of the insertion at the posterior cruciate ligament to the z-axis. The tibial x-axis was defined as the cross product of the y axis-and z-axis [7, 9] (Fig. 2). Pre-operative 3D planning was performed by reading implant computer-aided design (CAD) data from Evolution® (MicroPort Orthopaedics; Arlington, TN, USA) for all patients. During the pre-operative planning, the femoral components were replaced perpendicular to the mechanical axis, parallel to the surgical epicondylar axis and in some degrees of flexion (0–3°) to the 3D mechanical axis to avoid making an anterior notch. The tibial components were replaced perpendicular to the tibial anatomical axis. Posterior slopes were parallel to the lateral tibial plateau joint surface. Rotational alignments were matched to the line connecting the posterior cruciate ligament insertion and medial border to one third of the tibial tubercle [10] (Fig. 3).

**Fig. 4:**
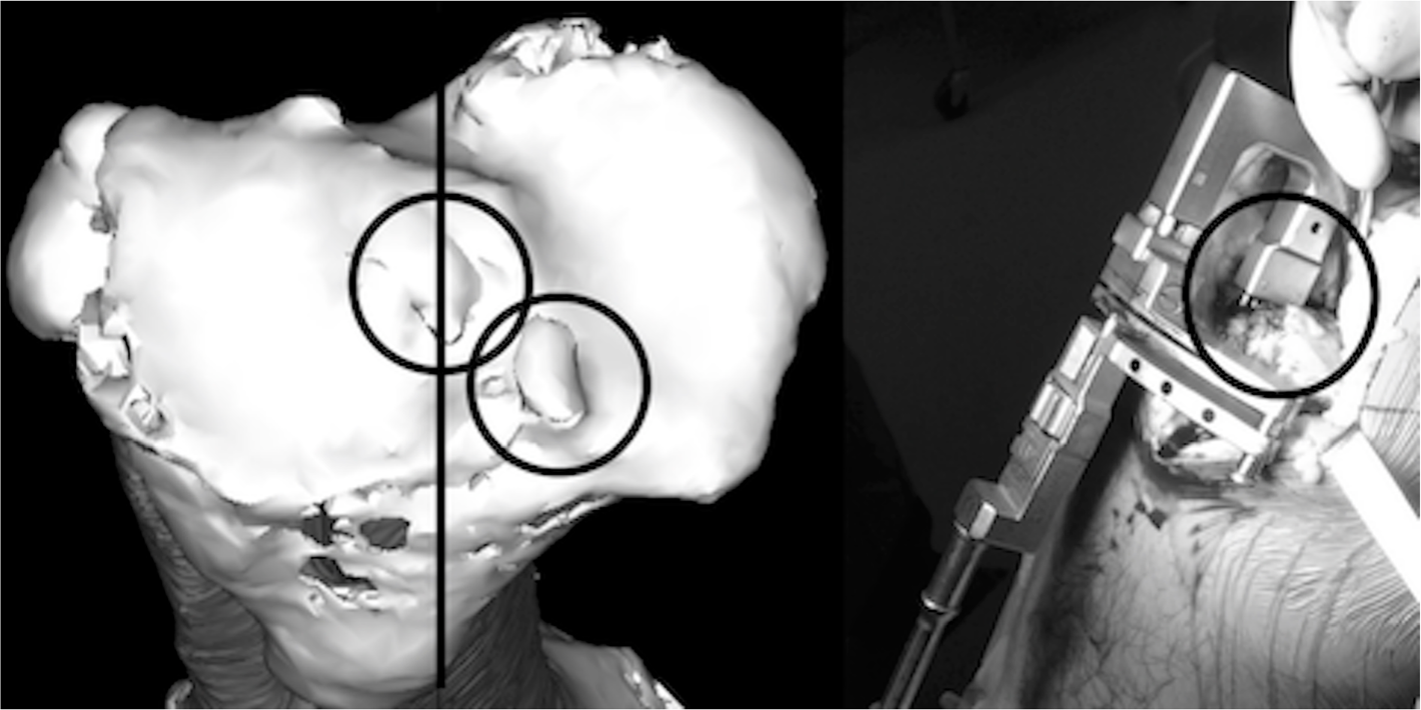
The extramedullary alignment rod was placed by referencing the three-dimensional (3D) model

Surgeries were performed by two orthopedic surgeons (OT and TM). Regarding the femoral cutting, an intramedullary alignment rod with a special jig that controlled the insertion point and depth of the alignment rod was used. At the proximal tibial cutting, a standard extramedullary alignment rod without any navigation system or special jig was used. The antero-posterior (AP) axis was confirmed by checking the 3D images captured during pre-operative planning. An extramedullary alignment rod was then placed along the AP axis with reference to the intercondylar eminence and osteophytes (Fig. 4). An image intensifier was used to confirm the extent of proximal tibial cutting and varus-valgus alignment. With the component in place, 3D images were used to check the relationship between the component and osteophyte related to the rotational alignment.

### Post-operative evaluation

**Fig. 5:**
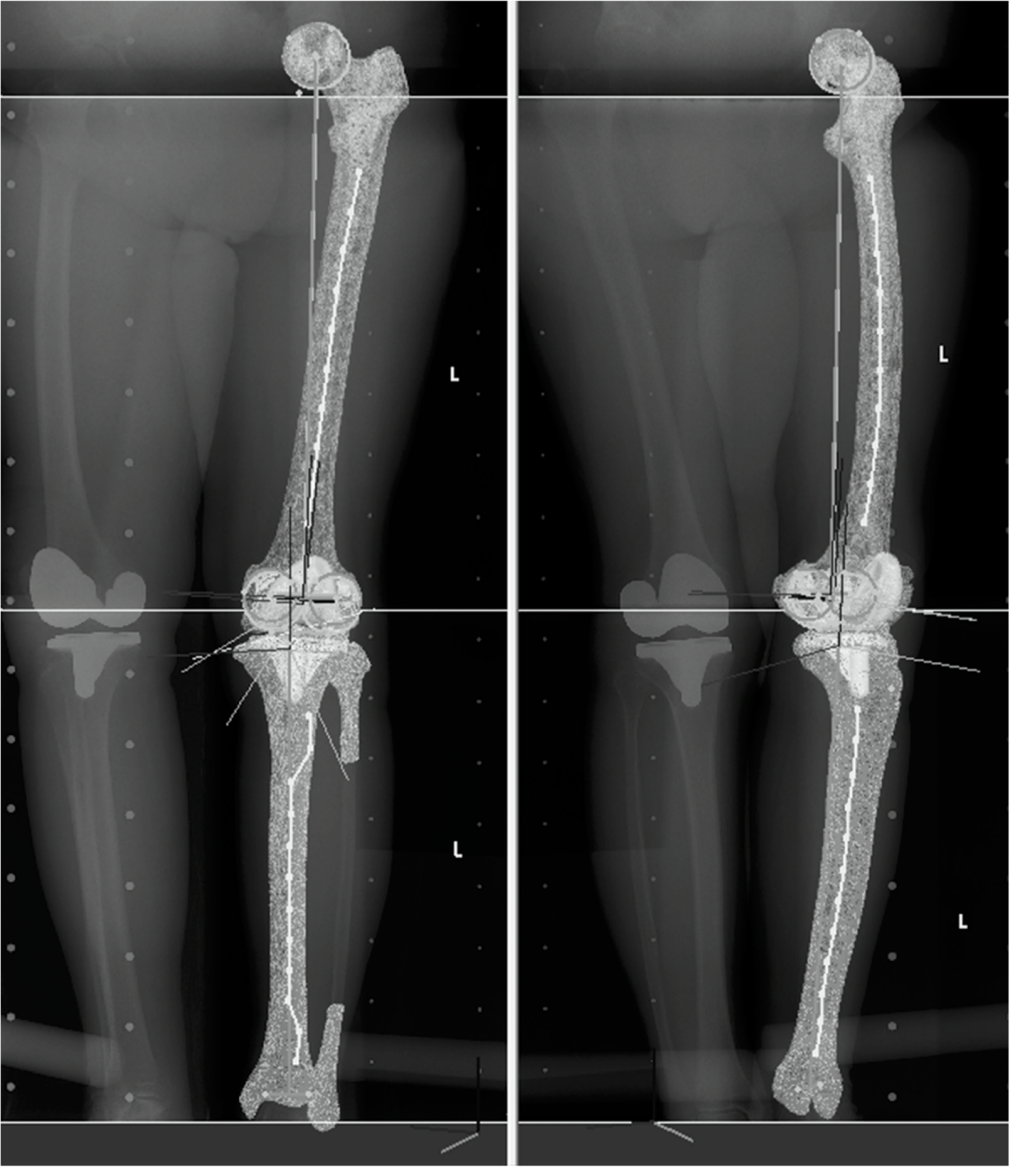
The three-dimensional (3D) post-operative evaluation of component position was performed using the same co-ordinates’ system as pre-operative planning. The information of the three-dimensional (3D) component position was obtained using 2D-to-3D image-matching technique for matching pre-operative computed tomography (CT) bone model and implant computer-aided design (CAD) model with post-operative biplanar CRs

A post-operative evaluation of component position was then performed. A 3D evaluation was performed by several orthopedic surgeons once per case. A 3D system to assess lower-extremity alignment, KneeCAS (LEXI, Inc., Tokyo, Japan) [6, 7, 11–14], was used for the post-operative evaluation using the same co-ordinates’ system as for the pre-operative planning (Fig. 5). This system consisted of a 2D-to-3D image-matching technique using CT and CR images. The patient’s whole-leg biplanar CRs (AP and 60° oblique view) were obtained 2 weeks after surgery. Then each implant CAD model of the femoral and tibial components was matched to the biplanar CR images. Pre-operatively constructed femoral and tibial 3D bone models that incorporated the co-ordinates’ system were also matched to the biplanar CR images. Through these processes, it was possible to obtain information about the relative position between the bone and the component, such as the femur and femoral component, or the tibia and tibial component, with the same co-ordinates’ system used during the pre-operative planning. Regarding the reproducibility of this system, the intra-observer reproducibility via the intra-class correlation coefficient (ICC) of the coronal and sagittal alignment were 0.99 and 1.00, respectively. The inter-observer reproducibility via the ICC of the coronal and sagittal alignment was 1.00 and 0.98, respectively [14].

**Fig. 6:**
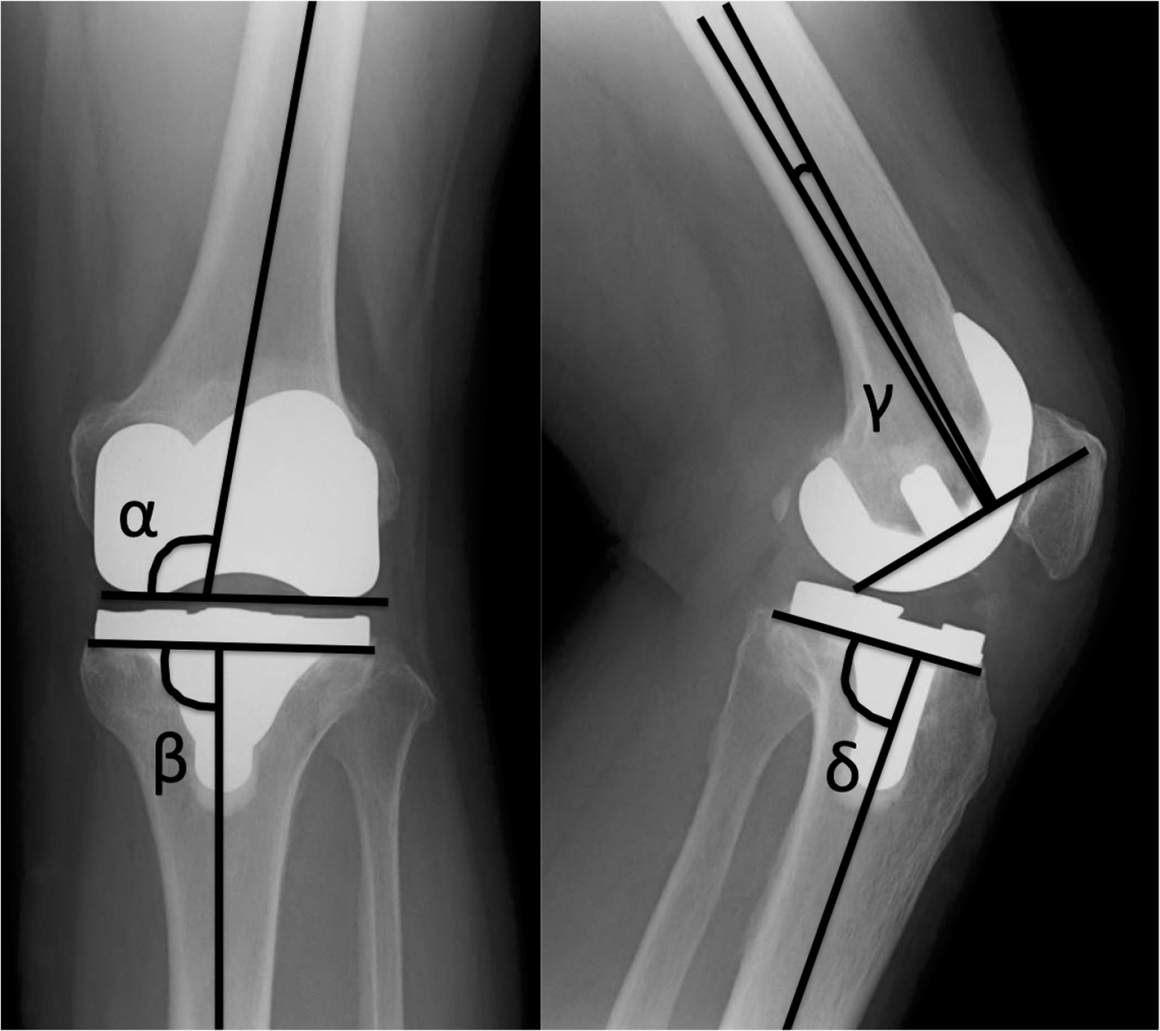
The two-dimensional (2D) post-operative evaluation of component position was performed using standard antero-posterior (AP) and lateral short-knee radiographs. In the AP view, component positions were measured using alpha (α) and beta (β) angles. In the lateral view, component positions were measured using gamma (γ) and delta (δ) angles

One orthopedic surgeon (OT) performed the 2D evaluation. Standard short-knee radiographs were used for the 2D post-operative evaluation (Fig. 6) [15]. Radiographs taken within 6 months of the surgery were selected and used with the components facing the true AP and true lateral. To evaluate inter-observer and intra-observer reproducibility of the 2D evaluation, two observers performed assessments for 30 subjects twice each. Intra-observer and inter-observer reproducibility of the coronal and sagittal alignment was examined using the ICC. The ICC values recorded by observer 1 were 0.744 and 0.789, whereas those recorded by observer 2 were 0.736 and 0.736, respectively. The ICC values between observers 1 and 2 were 0.757 and 0.736, respectively.

We investigated the differences between pre-operative planning and actual post-operative component positions, particularly with regard to the coronal and sagittal alignment. The differences were expressed in terms of absolute values. Then, that of an outlier—defined as a post-operative component position > ± 3° from the pre-operative planning—ratio was also investigated and compared with the results of the 3D and 2D evaluations.

### Statistical analysis

All data were expressed as mean ± standard deviation. Distribution and variance were examined using Shapiro-Wilk and Levene’s tests, respectively. Differences between the 3D and 2D evaluations were statistically analysed using the Wilcoxon signed-rank test. Differences in the ratio of outliers between the 3D and 2D evaluations were statistically analysed using the chi-squared test. The statistical significance level was set at *p* < 0.05. Statistical analyses were performed using IBM SPSS statistics version 21 (IBM Corp., Armonk, NY, USA).

## Results

The mean differences between the pre-operative planning and the actual post-operative coronal and sagittal alignments of the component are shown in Table 1. Regarding the femoral component’s sagittal alignment and the tibial component’s coronal and sagittal alignment, the difference in 3D evaluation was significantly higher than that of 2D evaluation (*p* = 0.013, = 0.003 and < 0.0001).

**Table 1:** The mean difference between pre-operative planning and post-operative component’s coronal and sagittal alignment

		3D evaluation	2D evaluation	*p* value
Femoral component	Coronal alignment (°)	**1.6** ± 1.4	**1.8** ± 1.2	0.083
		(0.03–7.48)	(0–6.0)	
	Sagittal alignment (°)	**2.6** ± 1.8	**1.9** ± 1.5	**0.013**
		(0.02–6.59)	(0–8.01)	
Tibial component	Coronal alignment (°)	**2.2** ± 1.8	**1.3** ± 1.2	**0.003**
		(0.03–6.54)	(0–5.01)	
	Sagittal alignment (°)	**3.2** ± 2.4	**1.8** ± 1.4	**< 0.001**
		(0.05–9.21)	(0–6.0)	

Values are presented as mean ± standard deviation (minimum to maximum). The statistical significance was set at *p* < 0.05

*3D* three-dimensional, *2D* two-dimensional

The outlier (> ± 3°) ratio of the actual post-operative component’s coronal and sagittal alignment are shown in Table 2. Regarding the femoral component’s sagittal alignment and the tibial component’s coronal and sagittal alignment, the outlier (> ± 3°) ratio for 3D evaluation was also significantly higher than that of the 2D evaluation (*p* < 0.001, = 0.009 and < 0.001).

**Table 2:** The outlier (over 3°) ratio of post-operative component’s coronal and sagittal alignment

		3D evaluation	2D evaluation	*p* value
Femoral component	Coronal alignment (%)	**13.8**	**7.7**	0.258
		(9 cases)	(5 cases)	
	Sagittal alignment (%)	**41.5**	**6.2**	**< 0.001**
		(27 cases)	(4 cases)	
Tibial component	Coronal alignment (%)	**24.6**	**7.7**	**0.009**
		(16 cases)	(5 cases)	
	Sagittal alignment (%)	**47.7**	**10.8**	**< 0.001**
		(31 cases)	(7 cases)	

Values are presented as number only. The statistical significance was set at *p* < 0.05

*3D* three-dimensional, *2D* two-dimensional

**Fig. 7:**
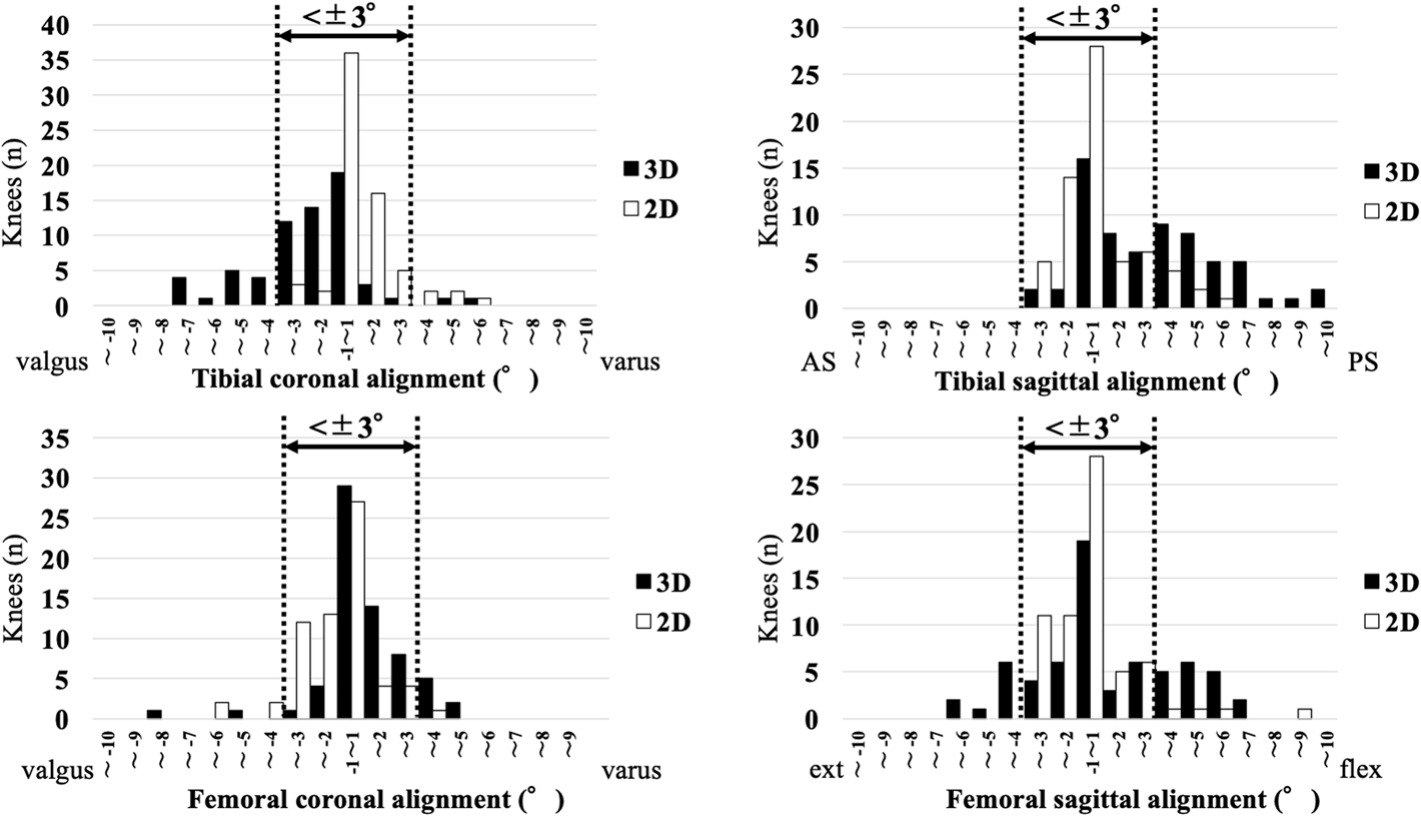
The distributions of the differences between pre-operative planning and the actual post-operative coronal and sagittal alignments of the component. *AS* anterior slope, *PS* posterior slope, e*xt* extension, *flex* flexion

The distributions of the differences between the pre-operative planning and the actual post-operative coronal and sagittal alignments of the component are shown in Fig. 7.

## Discussion

The most important finding of this study is that, compared to pre-operative planning, the difference in the 3D evaluation of post-operative component position was significantly greater than that in the 2D evaluation, even if the cases evaluated were identical.

There have been many studies on the post-operative 2D evaluation of component position. Cheng et al. [16] performed a meta-analysis of randomised controlled trials that evaluated the implant positioning through radiographs following TKA, comparing computer-assisted surgery and conventional techniques. Furthermore, in the conventional TKA group, a malalignment of > 3° in the coronal and sagittal plane was reportedly 15.8% and 41.3% in the femoral component and 8.6% and 21.8% in the tibial component, respectively. Many other studies have shown good results for the post-operative 2D evaluation of component position [17–22]. In our study, malalignment of > 3° in the coronal and sagittal plane was 7.7% and 6.2% in the femoral component and 7.7% and 10.8% in the tibial component, respectively, using the 2D evaluation. These results are comparable to, or better than, previous studies. However, Abu-Rajab et al. [1] and Park et al. [5] point out that standard AP knee radiographs have a problem with accuracy. Likewise, Hirschmann et al. reported that 2D CT has similar issues [2].

Many studies have investigated the post-operative 3D evaluation of component position [2, 23–25]. However, since the co-ordinates’ system of the post-operative evaluation is different from the co-ordinates’ system that was used for pre-operative planning, these evaluations do not accurately measure post-operative component positioning compared with pre-operative planning.

Recent studies have evaluated 3D component positioning using the same co-ordinates’ system as used in pre-operative planning. Ng et al. evaluated the post-operative 3D component position using 3D-CT [26]. They showed that a malalignment of > 2° of the tibial component in the coronal and sagittal planes in the conventional TKA group was 67% and 38%, respectively. In our study, malalignment of > 3° of the tibial component in the coronal and sagittal planes was also relatively high (24.6% and 47.7%, respectively) by the 3D evaluation, and significantly higher than that of the 2D evaluation (7.7% and 10.8%, respectively), even when the same cases were evaluated.

**Fig. 8:**
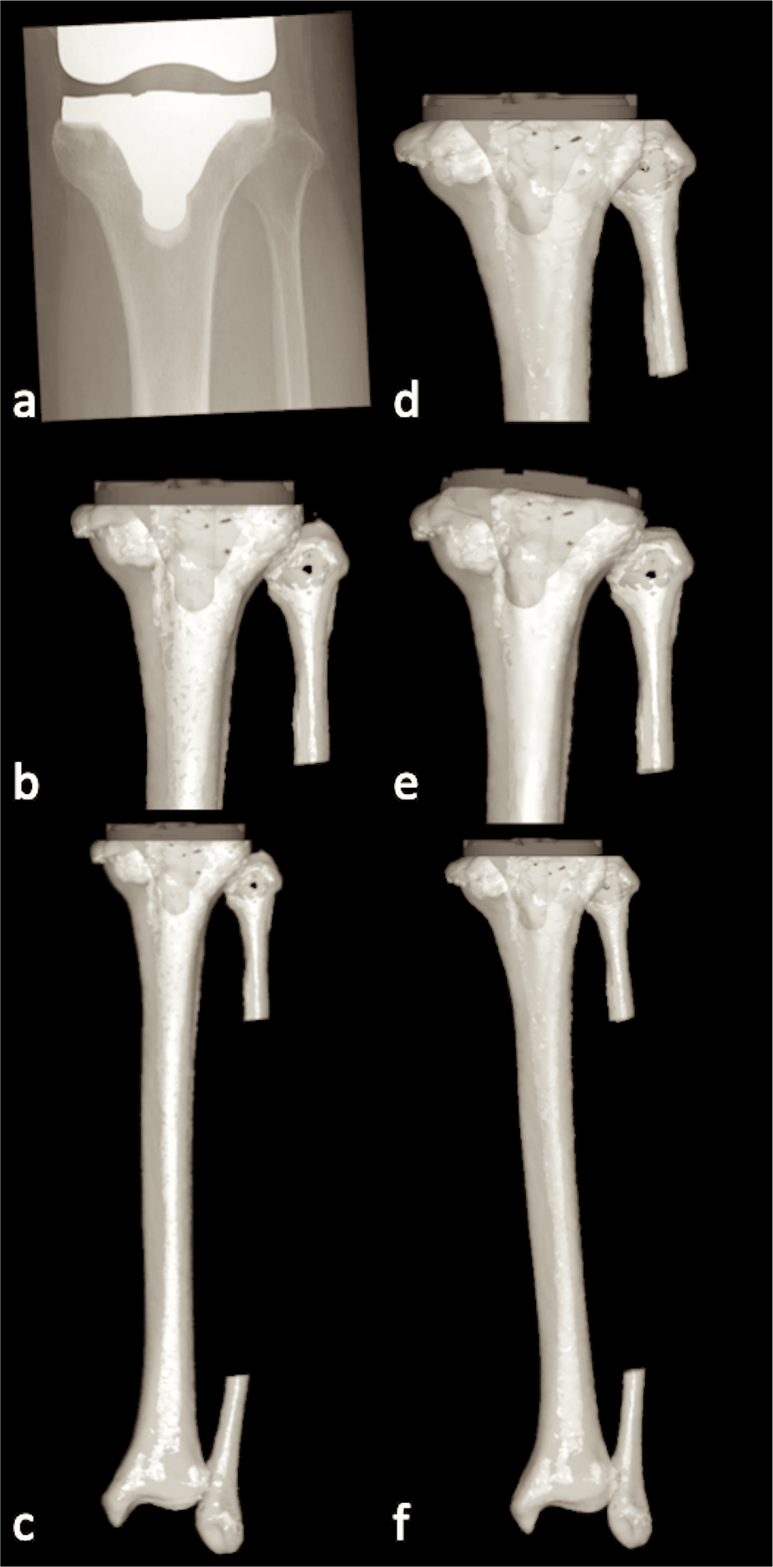
Pre-operative planning and post-operative evaluation. **a** Post-operative X-ray image. **b** Pre-operative planning of the tibial component. It can be seen that the rotational positional relationship between the tibia and fibula is different from the post-operative X-ray. **c** Pre-operative planning of the tibial component with the whole tibia. **d** Post-operative tibial component position. It can be seen that the rotational positional relationship between tibia and fibula is the same as the post-operative X-ray (**a**), but different from the pre-operative planning X-ray (**b**). **e** Post-operative tibial component positioning presented by the co-ordinates’ system as pre-operative planning. It can be seen that the tibial component is placed in more valgus, posterior slope and internal rotated position than the pre-operative planning (**b**). **f** Post-operative tibial component position with the whole tibia

It should be noted that the results changed depending on different co-ordinates’ systems. During pre-operative planning, co-ordinates’ systems are constructed for each of the femoral and tibial bone models, and the components positioned within them. However, during post-operative evaluation using 2D standard short-knee radiography, radiographs of the components facing true AP and true lateral planes must be used. These are not necessarily true AP and lateral views against the femur and tibia. In other words, different co-ordinates’ systems are used between pre-operative planning and post-operative evaluation (Fig. 8). As a result, this 2D evaluation could not be precise. In this point, Mizu-Uchi et al. also showed that the discrepancy between the 2D and 3D evaluations of post-operative alignment for TKA was 1.0° ± 0.9° (0.1–3.4°). They suggested that it is important to measure the post-operative alignment in three dimensions for an exact evaluation, whereas 3D analysis is also necessary to assess the accuracy of the navigation system [27].

It is expected that the discrepancy between the 2D and 3D evaluations occurs in cases that have a large error of rotational alignment. In our study, the coronal and sagittal alignment of the tibial components showed significant differences between the 2D and 3D evaluations, whereas the coronal alignment of the femoral component did not show a significant difference between the 2D and 3D evaluations. Regarding the tibial component, a standard extramedullary alignment rod without any navigation system or special jig was used. Therefore, errors of rotational alignment occurred at the timing of not only proximal tibial cutting but also cementing and these errors affected the coronal and sagittal alignment. Then, the 3D evaluation might detect these errors more than the 2D evaluation. Regarding the femoral component, an intramedullary alignment rod with a special jig was used. Therefore, errors during femoral cutting were small including the rotational alignment and the coronal alignment of the femoral component did not show a significant difference between 2D and 3D evaluations. Regarding the sagittal alignment of the femoral component, it was considered that there was an error during cementing, and the 3D evaluation could detect it more than the 2D evaluation.

Our study raised the possibility that 2D post-operative evaluation underestimates the differences between pre-operative planning and the actual post-operative component positions. Recently, many studies have reported the occurrence of unexplained knee pain following TKA, although post-operative alignment showed no problems through 2D evaluation [28–30]. In these cases, malalignment may exist through strict 3D evaluation. This may be one of the reasons why patients with unexplained knee pain exist, even though they do not have any problems of alignment, based on 2D evaluation.

This study has certain limitations. First, the sample size of patients was small. However, there was enough power for the results to be considered statistically significant. Based on a sample size calculation (α error, 0.05; 1 – *β*, error 0.80), 42 knees were estimated to detect significant differences between the 3D evaluation and 2D evaluation. This study included a sufficient sample size (65 knees). Second, the standard short-knee radiographs were used for the 2D post-operative evaluation. A full-length X-ray could be used for more accurate evaluations than standard short-knee radiographs, and different results could have been consequently obtained. However, in this study, standard short-knee radiographs were used to emphasise the difference between the 2D and 3D evaluations.

## Conclusions

This study demonstrated that differences between pre-operative planning and post-operative component alignment in the 3D evaluation of component positions were significantly higher than those of 2D evaluation, even if the same cases were evaluated. Two-dimensional evaluation may mask or underestimate the post-operative implant malposition that could potentially induce modern clinical problems such as unexplained knee pain in TKA. Three-dimensional evaluation using the same co-ordinates’ system as for pre-operative planning is necessary to accurately evaluate the post-operative component positions.
